# Valorization of Hom Thong Banana Peel (*Musa* sp., AAA Group) as an Anti-Melanogenic Agent Through Inhibition of Pigmentary Genes and Molecular Docking Study

**DOI:** 10.3390/ijms252313202

**Published:** 2024-12-08

**Authors:** Pichchapa Linsaenkart, Wipawadee Yooin, Supat Jiranusornkul, Korawan Sringarm, Chaiwat Arjin, Pornchai Rachtanapun, Kittisak Jantanasakulwong, Juan M. Castagnini, Warintorn Ruksiriwanich

**Affiliations:** 1 Department of Pharmaceutical Sciences, Faculty of Pharmacy, Chiang Mai University, Chiang Mai 50200, Thailand; pichchapa_li@cmu.ac.th (P.L.); wipawadee.y@cmu.ac.th (W.Y.); supat.jira@cmu.ac.th (S.J.); 2 Cluster of Valorization and Bio-Green Transformation for Translation Research Innovation of Raw Materials and Products, Chiang Mai University, Chiang Mai 50200, Thailand; korawan.s@cmu.ac.th; 3 Cluster of Agro Bio-Circular-Green Industry (Agro BCG), Chiang Mai University, Chiang Mai 50200, Thailand; pornchai.r@cmu.ac.th (P.R.); kittisak.jan@cmu.ac.th (K.J.); 4 Department of Animal and Aquatic Sciences, Faculty of Agriculture, Chiang Mai University, Chiang Mai 50200, Thailand; chaiwat.arjin@cmu.ac.th; 5 School of Agro-Industry, Faculty of Agro-Industry, Chiang Mai University, Chiang Mai 50100, Thailand; 6 Research Group in Innovative Technologies for Sustainable Food (ALISOST), Department of Preventive Medicine and Public Health, Food Science, Toxicology and Forensic Medicine, Faculty of Pharmacy, Universitat de València, Avenida Vicent Andrés Estellés s/n, 46100 Burjassot, Spain; juan.castagnini@uv.es

**Keywords:** anti-melanogenesis, banana peel, Hom Thong, rosmarinic acid, tyrosinase

## Abstract

Prolonged and unprotected exposure to the environment explicitly influences the development of hyperpigmented lesions. The enzyme tyrosinase (TYR) is a key target for regulating melanin synthesis. Several bioactive compounds derived from plant extracts have been found to possess potent anti-melanogenesis properties against TYR. In particular, the potential of banana peels from various varieties has garnered interest due to their application in skin hyperpigmentation treatment. A molecular docking study demonstrated interactions between rosmarinic acid, which is predominantly found in all Hom Thong peel extracts, and the active site of TYR (PDB ID: 2Y9X) at residues HIS263, VAL283, SER282, and MET280, with the lowest binding energy of −5.05 kcal/mol, showing the strongest interaction. Additionally, Hom Thong banana peels are rich in phenolic compounds that could inhibit melanin content and tyrosinase activity in both human and mouse melanoma cells. These effects may be attributed to the suppression of gene expression related to melanogenesis, including the regulator gene *MITF* and pigmentary genes *TYR*, *TRP-1*, and *DCT*, indicating effects comparable to those of the standard treatment groups with arbutin and kojic acid. Our findings indicated the potential of Hom Thong peel extracts as anti-melanogenic agents.

## 1. Introduction

Skin imbalance may arise from an impaired antioxidant system and the upregulation of inflammatory cytokines in keratinocytes, which contribute to increased melanogenesis in surrounding melanocytes. Chronic exposure to environmental factors manifests as signs of aging, particularly hyperpigmented lesions [1,2]. Previous studies have reported psychological consequences for people affected by melasma. Among these patients, anxiety, depression, and low self-esteem are commonly observed psychological conditions [3,4]. Additionally, there is an established relationship between the presence of pre-exciting hyperpigmented lesions and the development of cutaneous melanoma. To illustrate, lentigo maligna, a rare type of melanoma, arises from UV-exposed skin lesions [5,6].

Melanin pigments are biosynthesized from the substrates L-tyrosine and L-3,4-dihydroxyphenylalanine (L-DOPA). Tyrosinase (TYR), the key melanogenic enzyme, catalyzes the conversion of both substrates to L-dopachrome, a step recognized as the rate-limiting reaction in melanin production. Other members of the tyrosinase enzyme family, including tyrosinase-related protein-1 (TRP-1) and tyrosinase-related protein-2, also known as dopachrome tautomerase (TRP-2 or DCT), are subsequently involved in the final steps of melanin formation. The expression of these pigmentary genes is regulated by the transcription factor known as microphthalmia-associated transcription factor (*MITF*). The activation of *MITF* may result in the upregulation of other pigment-related genes [2].

The activity of tyrosinase is a key factor in regulating melanogenesis. The active site of tyrosinase is highly conserved, containing two copper atoms (CuA and CuB), coordinated with six histidine residues [7,8]. Molecular docking analysis revealed that tyrosinase interacts with the hydroxyl group of the L-tyrosine substrate near the CuB atom and one of the histidine residues. Additionally, the phenol group of the L-DOPA ligand is positioned near the CuB atom. The catalytic mechanism of tyrosinase involves the monophenolase reaction, in which L-tyrosine is oxidized by tyrosinase to form L-DOPA. This is subsequently catalyzed to dopaquinone (DQ) in the diphenolase reaction [9,10].

Triple combination therapy, consisting of hydroquinone, retinoids, and topical steroids, is commonly recommended for melasma and hyperpigmentation treatment [11,12]. The action of hydroquinone involves inducing epidermal cell death but poses a risk of skin cancer, limiting its use strictly to medical purposes [13]. Vitamin A derivatives, such as tretinoin, tazarotene, or adapalene, act primarily by inhibiting melanosome transfer and promoting exfoliation. However, the use of these compounds frequently leads to skin hypersensitivity [11,14]. Consequently, various cosmeceutical ingredients have been developed, either through structural modification or derived from natural resources, to reduce toxicity to skin cells. For instance, arbutin, a derivative of hydroquinone extracted from bearberry leaves, and kojic acid, a byproduct of fungus, are known to inhibit tyrosinase activity while also providing antioxidant benefits [14]. Numerous studies focus on evaluating medicinal plants that contribute to regulating melanogensis for the treatment of dermatological problems [15,16]. With regard to these, the discovery of bioactive compounds derived from natural resources may offer effective treatment for hyperpigmented lesions with fewer undesirable side effects.

Bananas, fruits from the *Musaceae* family, are widely consumed globally and are predominantly produced in regions such as Asia, Latin America, and Africa. Various banana species are consumed either as fresh fruit or incorporated into a variety of culinary preparations, such as desserts or beverages [17]. Moreover, in Thai cuisine, banana flowers and pseudostems are used as ingredients in curries, side dishes, or stir-fries [18]. Banana peels, the inedible parts, are recognized as a major waste after food processing, with approximately 40 million tons generated annually [19]. Previous studies have reported that they are rich in several groups of bioactive compounds, including flavonols, flavanones, tannin, hydroxycinnamic acid derivatives, as well as carotenoids. These molecules have potential for antioxidant, anti-inflammatory, antimicrobial, or hypoglycemic effects [17,20].

Interestingly, the peels of *Musa sapientum* ABB group ‘Kluai Namwa’, *M. sapientum* AA group ‘Leb Mu Nang’, and sucrier banana have been shown to exhibit anti-melanogenic effects. These effects are potentially linked to the phenolic compounds and carotenoids found in banana peels [21,22,23]. The variety of banana species and their geographical origins have a significant impact on the characteristics and bioactive composition of bananas. In addition, to date, no study has accurately identified the specific compounds present in banana peels that are responsible for modulating the melanogenesis pathway.

In this study, we identified the bioactive compounds from the peels of the Hom Thong banana, a popular species that is also widely used in food. The polyphenols in the crude extracts were elucidated using liquid chromatography coupled with mass spectrometry. All extracts and bioactive compounds found in Hom Thong banana peels were screened for anti-melanogenic properties using mushroom tyrosinase. To further understand the interaction with this enzyme, a molecular docking analysis was conducted to predict how these compounds bind to melanogenic enzyme. Additionally, melanoma cell lines were used to assess the effects of the extracts and their potential compounds on cell viability, melanin production within the cells, and intracellular tyrosinase activity, indirectly determined by measuring the amount of L-dopachrome formation. Ultimately, the expression levels of key genes involved in melanogenesis, including *MITF*, *TYR*, *TRP-1*, and *DCT*, were analyzed.

## 2. Results

### 2.1. Extraction Yields and Phenolic Composition

In this study, the extraction yields resulting from the same maceration technique with different extraction solvents were 8.25%, 22.05%, and 10.65% *w*/*w* based on dry samples for aqueous, 50% ethanolic, and 95% ethanolic extraction, respectively. The characteristics of the polyphenol components identified by the LC-ESI/MS method are demonstrated in Table 1. The aqueous extract of banana peel primarily contains catechin and *o*-coumaric acid. In contrast, ferulic acid is exclusively found in 50% ethanolic extract. Rosmarinic acid is predominantly present in the 95% ethanolic extract.

### 2.2. Effects of Banana Peel Extracts on Mushroom Tyrosinase Activity

A preliminary screening of anti-melanogenic activity was performed by measuring the levels of L-dopachrome formation resulting from the reaction between the substrates, L-tyrosine or L-DOPA, and mushroom tyrosinase. The results were expressed as IC_50_ values, as shown in Table 2. The IC_50_ values of each banana peel extract for monophenolase and diphenolase reactions, respectively, were in ascending order: Water (5.56 ± 0.46 and 17.35 ± 3.24 mg/mL), 50EtOH (2.33 ± 0.23 and 2.70 ± 0.07 mg/mL), and 95EtOH (0.92 ± 0.15 and 2.01 ± 0.17 mg/mL). The anti-melanogenic activity of 50EtOH was comparable to that of commercial tyrosinase inhibitors against the diphenolase activity of mushroom tyrosinase. Notably, 95EtOH extract exhibited stronger inhibition of the diphenolase reaction, being approximately 1.71 times more effective than arbutin and 1.73 times more effective than kojic acid. Among the pure phenolic compounds, *o*-coumaric acid exhibited the most potent inhibitory effect on the monophenolase reaction (IC_50_ = 1.39 ± 0.03 mg/mL). Notably, *o*-coumaric acid, ferulic acid, and rosmarinic acid demonstrated strong inhibitory effects on the diphenolase reaction catalyzed by mushroom tyrosinase, with IC_50_ values of 4.19 ± 0.15, 3.93 ± 0.09, and 3.63 ± 0.08 mg/mL, respectively. These values were comparable to those of tyrosinase inhibitors, arbutin and kojic acid.

### 2.3. Interaction of Bioactive Compounds from Banana Peel Extracts with Mushroom Tyrosinase

A molecular docking simulation was conducted to elucidate interactions and binding affinities of bioactive compounds identified from Hom Thong banana peel extracts with mushroom tyrosinase (PDB ID: 2Y9X). Tropolone, the native ligand, was redocked into the active site of *Agaricus bisporus* mushroom tyrosinase for method validation (Figure S1a). All 100 conformations were part of the same cluster, with the lowest-energy conformation exhibiting a binding energy of −4.41 kcal/mol. The binding energy of each ligand towards mushroom tyrosinase, obtained from AutoDock, is summarized in Table 3. A more negative binding free energy corresponds to a stronger binding affinity with the target enzyme [24]. The results indicated that the substrate L-DOPA had the lowest binding energy (−5.37 kcal/mol) towards mushroom tyrosinase, followed by L-tyrosine (−5.18 kcal/mol). Among the phenolic compounds, (+)-catechin and *(R)*-rosmarinic acid displayed stronger binding affinities, with binding energy values of −5.07 and −5.05 kcal/mol, respectively, compared to arbutin and kojic acid (−4.06 and −3.60 kcal/mol, respectively). 

**Figure 1:**
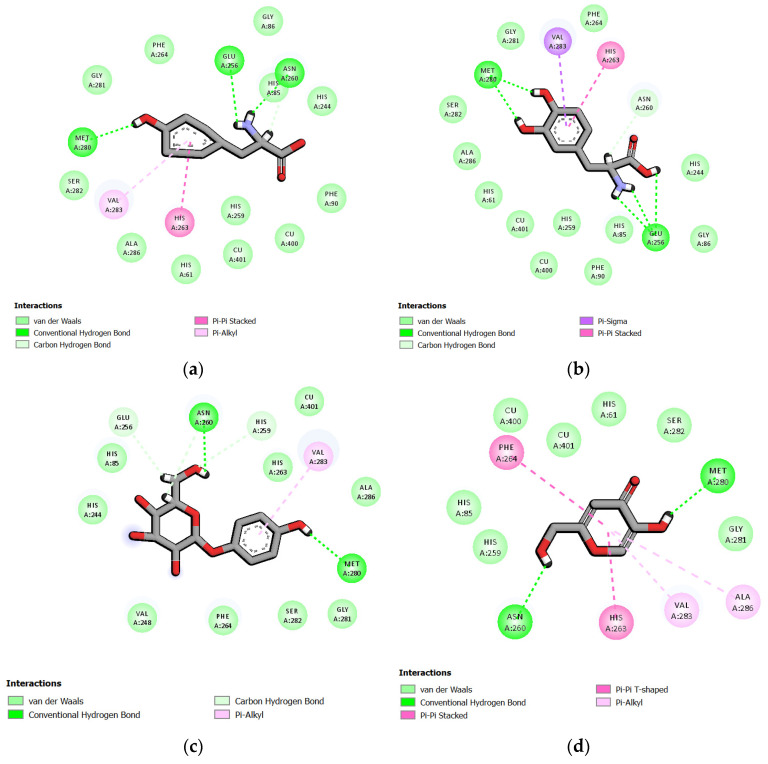
The molecular interaction profile of mushroom tyrosinase (PDB ID: 2Y9X) and the ligands after molecular docking studies binding poses of (**a**) L-tyrosine; (**b**) L-DOPA; (**c**) β-arbutin; and (**d**) kojic acid visualized by the BIOVIA Discovery Studio Visualizer.

**Figure 2:**
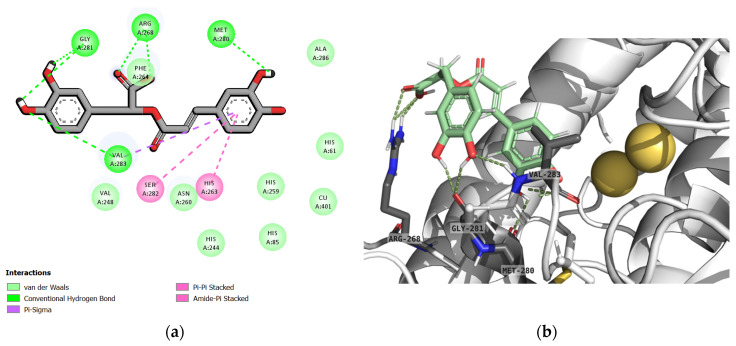
The molecular interaction profile of (**a**) 2D and (**b**) 3D structures of *(R)*-rosmarinic acid towards mushroom tyrosinase (PDB ID: 2Y9X) visualized by the BIOVIA Discovery Studio Visualizer and PyMOL, respectively.

According to the results from the mushroom tyrosinase inhibitory assay, all extracts contained *(R)*-rosmarinic acid, especially 95EtOH extract exhibiting the lowest IC_50_ values against mushroom tyrosinase. Thus, the interactions between the substrates (L-tyrosine and L-DOPA), standard tyrosinase inhibitors (β-arbutin and kojic acid), and the potential bioactive compound, *(R)*-rosmarinic acid, with mushroom tyrosinase are represented in Figure 1. The interactions of L-tyrosine, L-DOPA, kojic acid, and *(R)*-rosmarinic acid showed that all ligands possessed aromatic rings capable of forming π-bonds with amino acids in the active site, specifically HIS263 and VAL283. However, the aromatic ring of β-arbutin interacted only with VAL283 through π-bonding. Interestingly, *(R)*-rosmarinic acid also formed a π-bond with SER282. Regarding hydrogen bonding, all compounds were found to form hydrogen bonds with MET280. Furthermore, *(R)*-rosmarinic acid formed more hydrogen bonds than both substrates, L-tyrosine and L-DOPA. The 3D structure visualized by PyMOL in Figure 2 illustrates the arrangement of *(R)*-rosmarinic acid in the active site, revealing that the structure folded to fit into the space and formed hydrogen bonds, consistent with the 2D image.

To further confirm these findings, the potential of all samples was tested using melanoma cell models, including G-361 human melanoma cells and B16 mouse melanoma cells, which provide reliable and reproducible methods for quantifying melanin [13].

### 2.4. Banana Peel Extracts Inhibit Melanogenesis in Melanoma Cells

#### 2.4.1. Inhibitory Effects of Banana Peel Extracts on Intracellular Melanin Content and Tyrosinase Activity

**Figure 3:**
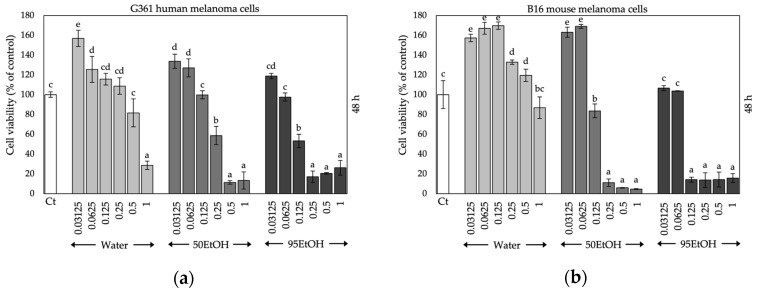

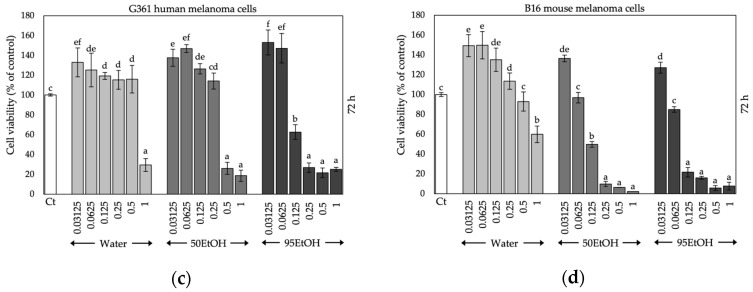
Effects of Hom Thong banana peel extracts on cell viability in (**a**) G361 human melanoma cells at 48 h; (**b**) B16 mouse melanoma cells at 48 h; (**c**) G361 human melanoma cells at 72 h; and (**d**) B16 mouse melanoma cells at 72 h. Data are expressed as the mean ± SD. Significant differences between samples are indicated by different letters (a, b, c, d, e, and f) with *p* < 0.05. Ct: control, Water: aqueous extract of Hom Thong banana peel, 50EtOH: 50% ethanolic extract of Hom Thong banana peel, 95EtOH: 95% ethanolic extract of Hom Thong banana peel.

Initially, cells were exposed to various concentrations of the extracts (0.03125–1 mg/mL) to determine the optimal concentration that exhibited no cytotoxicity before proceeding with further experiments. The results indicated that all extracts did not significantly affect cell viability (cell viability remained above 80%) at concentrations up to 0.0625 mg/mL for 48 and 72 h of treatment (Figure 3). Consequently, a concentration of 0.0625 mg/mL was selected for use in subsequent experiments with G361 human melanoma cells and B16 mouse melanoma cells.

**Figure 4:**
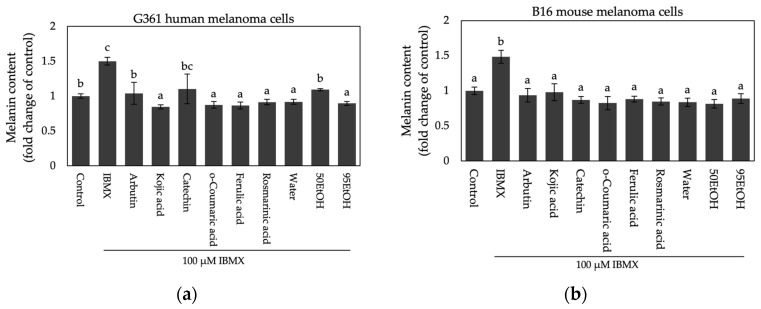

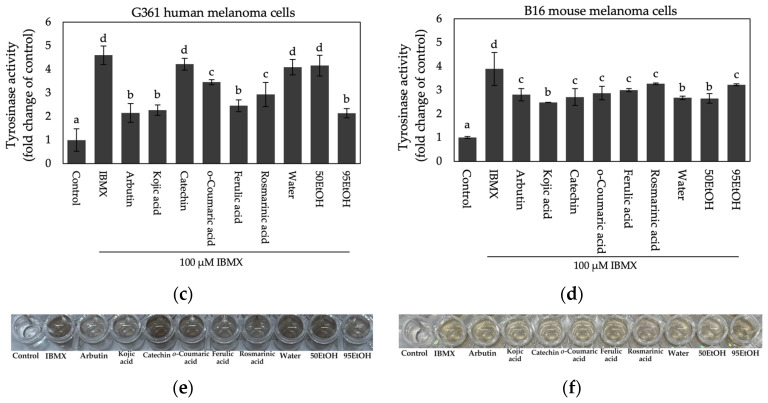
Effects of Hom Thong banana peel extracts and their bioactive compounds on (**a**) melanin content in G361 human melanoma cells; (**b**) melanin content in B16 mouse melanoma cells; (**c**) tyrosinase activity in G361 human melanoma cells; and (**d**) tyrosinase activity in B16 mouse melanoma cells. Figures of L-dopachrome formation in (**e**) G361 human melanoma cells and (**f**) B16 mouse melanoma cells. Data are expressed as the mean ± SD. Significant differences between samples are indicated by different letters (a, b, c, and d) with *p* < 0.05. Water: aqueous extract of Hom Thong banana peel, 50EtOH: 50% ethanolic extract of Hom Thong banana peel, 95EtOH: 95% ethanolic extract of Hom Thong banana peel.

Then, intracellular melanin content and tyrosinase activity assays were investigated in both cell types. Melanin content was measured by dissolving cell pellets in an alkaline solution. As shown in Figure 4a,b, induction with IBMX upregulated melanin produced in cell pellets, with approximately 1.50 ± 0.06- and 1.49 ± 0.09-fold change in G361 and B16 melanoma cells, respectively. Pre-treatment with Hom Thong banana peel extracts and their phenolic compounds inhibited melanin production in both cell types, showing results comparable to those of groups treated with the standard tyrosinase inhibitors, arbutin and kojic acid. For cellular tyrosinase activity, L-DOPA substrate was incubated with the supernatant from lysed cell pellets to measure L-dopachrome formation. Notably, exposure to IBMX significantly increased L-dopachrome formation compared to untreated control cells, with fold changes of 4.60 ± 0.40 and 3.89 ± 0.69 in human and mouse melanoma cells, respectively (Figure 4c,d). In the G361 human melanoma model, only 95EtOH significantly reduced tyrosinase activity (relative fold change of 2.14 ± 0.19), reaching levels comparable to arbutin and kojic acid treatment (relative fold changes of 2.15 ± 0.40 and 2.27 ± 0.22, respectively), as illustrated in the 96-well plate assay results shown in Figure 4e. In the case of mouse melanoma cells, all treatments inhibited L-dopachrome formation, with water- and 50EtOH-treated groups showing particularly strong effects (relative fold changes of 2.67 ± 0.07 and 2.65 ± 0.20, respectively), as represented in the 96-well plate after incubation with L-DOPA (Figure 4f).

#### 2.4.2. Inhibitory Effects of Banana Peel Extracts on the Expression of Transcription Factor MITF and Pigmentary Genes

**Figure 5:**
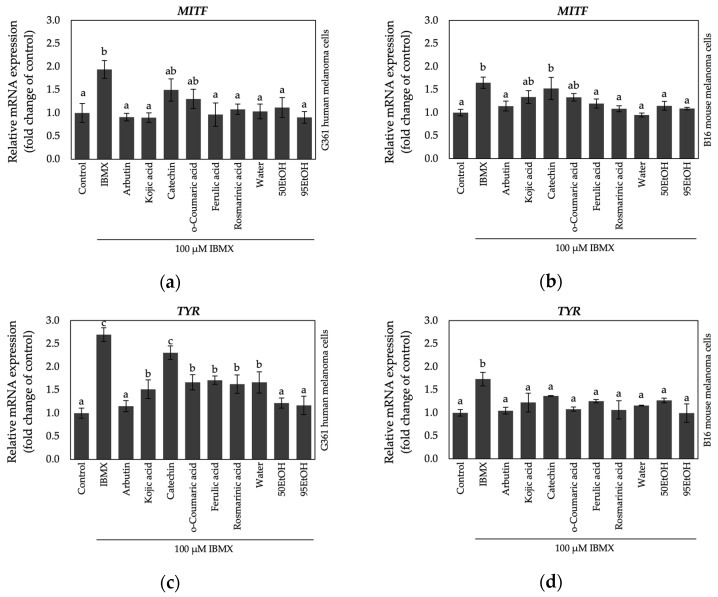

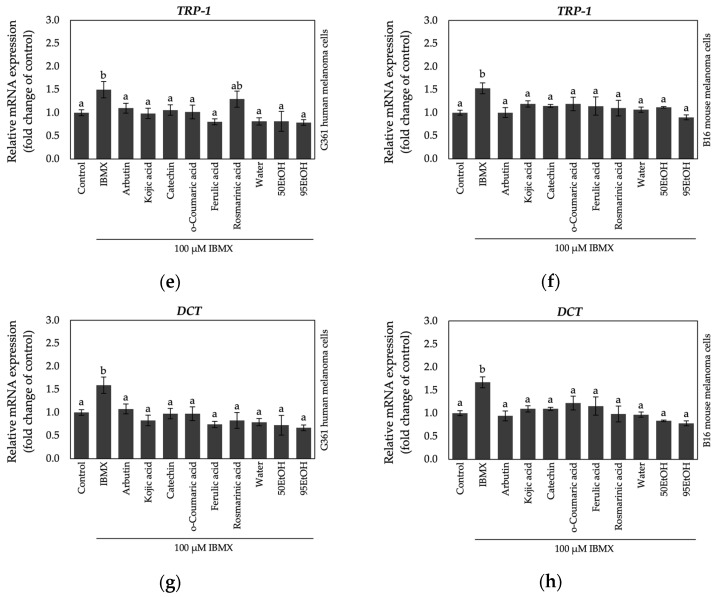
Effects of Hom Thong banana peel extracts and their bioactive compounds on relative gene expression of (**a**) *MITF* in G361 human melanoma cells; (**b**) *MITF* in B16 mouse melanoma cells; (**c**) *TYR* in G361 human melanoma cells; (**d**) *TYR* in B16 mouse melanoma cells; (**e**) *TRP-1* in G361 human melanoma cells; (**f**) *TRP-1* in B16 mouse melanoma cells; (**g**) *DCT* in G361 human melanoma cells; and (**h**) *DCT* in B16 mouse melanoma cells. Data are expressed as the mean ± SD. Significant differences between samples are indicated by different letters (a, b, and c) with *p* < 0.05. Water: aqueous extract of Hom Thong banana peel, 50EtOH: 50% ethanolic extract of Hom Thong banana peel, 95EtOH: 95% ethanolic extract of Hom Thong banana peel.

To make a further determination, the levels of gene expression associated with melanogenesis were analyzed. The relative mRNA expression levels of *MITF*, *TYR*, *TRP-1*, and *DCT* are illustrated in Figure 5. In G361, human melanoma cells stimulated with the cyclic adenosine monophosphate (cAMP) elevator, 3-isobutyl-1-methylxanthine (IBMX), the relative mRNA levels of *MITF*, *TYR*, *TRP-1*, and *DCT* were significantly increased to 1.94 ± 0.19, 2.70 ± 0.15, 1.50 ± 0.18, and 1.60 ± 0.31, respectively, compared to those of the untreated cells. Along with the results observed in B16 mouse melanoma cells, the relative gene expression levels of *MITF*, *TYR*, *TRP-1*, and *DCT*, after exposure to IBMX treatment, were significantly upregulated to 1.65 ± 0.12, 1.73 ± 0.15, 1.53 ± 0.12, and 1.67 ± 0.13, respectively, compared to the control group.

The key regulator gene involved in melanin biosynthesis is *MITF*. The downregulation of *MITF* may contribute to the reduction in other pigment-related genes [25]. As shown in Figure 5a, ferulic acid and rosmarinic acid presented strong inhibitory effects on *MITF* expression, with relative expression values of 0.96 ± 0.25 and 1.08 ± 0.11, respectively. Remarkably, all types of banana peel extracts, including Water, 50EtOH, and 95EtOH, significantly suppressed transcriptional *MITF* gene expression to 1.03 ± 0.16, 1.12 ± 0.21, and 0.90 ± 0.13, respectively, in IBMX-induced G361 cells. These results were comparable to those of the reference compounds arbutin (0.91 ± 0.08) and kojic acid (0.90 ± 0.11). Similarly, in B16 cells, both ferulic acid and rosmarinic acid also inhibited *MITF* expression, with relative levels of 1.12 ± 0.10 and 1.08 ± 0.07, respectively, which were comparable to those observed with arbutin treatment (1.14 ± 0.11) (Figure 5b). Interestingly, *MITF* expression levels also were significantly suppressed to 0.95 ± 0.04, 1.15 ± 0.09, and 1.09 ± 0.03 after treatment with Water, 50EtOH, and 95EtOH, respectively.

Tyrosinase, encoded by *TYR*, is the enzyme responsible for the rate-limiting step in melanogenesis and serves as the primary target for anti-melanogenic agents [14]. As shown in Figure 5c, *TYR* mRNA levels were reduced to 1.67 ± 0.16, 1.71 ± 0.09, and 1.63 ± 0.20 following treatment with *o*-coumaric acid, ferulic acid, and rosmarinic acid, respectively, in comparison to *TYR* expression in the kojic acid-treated group (1.52 ± 0.20). Notably, 50EtOH and 95EtOH exhibited more potent *TYR* inhibition than these polyphenol compounds, with relative expression levels of 1.22 ± 0.11 and 1.17 ± 0.20, respectively. These effects were comparable to those observed in the arbutin treatment group (1.15 ± 0.12). Additionally, all three banana peel extracts, along with their phenolic constitutes, significantly decreased *TYR* mRNA expression levels in the B16 mouse melanoma model (Figure 5d).

Tyrosinase-related proteins type 1 and 2, encoded correspondingly by *TRP-1* and *DCT*, also play crucial roles in melanin synthesis following L-dopachrome formation. These melanogenic enzymes are essential for completing the synthesis pathway of eumelanin, or black-brown pigments [26]. As depicted in Figure 5e, catechin, *o*-coumaric acid, ferulic acid, and all extracts were able to inhibit *TRP-1* expression to levels comparable to those observed with the depigmenting agents, arbutin and kojic acid (1.10 ± 0.11 and 0.99 ± 0.11, respectively) in IBMX-induced G361 cells. Moreover, all treatment groups suppressed the mRNA levels of *DCT*, as illustrated in Figure 5g. These findings were consistent with the results obtained from B16 mouse melanoma cells. As shown in Figure 5f,h, all depigmenting agents, phenolic compounds, and banana peel extracts significantly decreased the relative expression level of both *TRP-1* and *DCT* in IBMX-stimulated B16 cells.

## 3. Discussion

**Figure 6:**
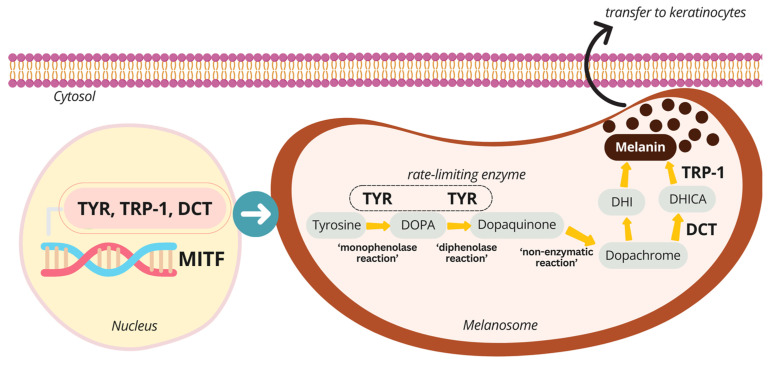
The scheme summarizes melanin biosynthesis.

Generally, melanin synthesis begins with the conversion of the initial substrate, L-tyrosine, to L-DOPA, which is then oxidized to DQ and subsequently to L-dopachrome. TYR is the primary enzyme responsible for the rate-limiting step in this process. Afterward, L-dopachrome is spontaneously converted to 5,6-dihydroxyindole (DHI), leading to melanin formation. In addition, L-dopachrome may gradually be converted to 5,6-dihydroxyindole-2-carboxylic acid (DHICA), with melanin eventually formed through enzymatic reactions facilitated by DCT and TRP-1, respectively [26] (Figure 6). The TYR family, which includes TYR, TRP-1, and DCT, are metalloenzymes with approximately 40% homology in their amino acid sequences. A key structural region is the intramelanosomal domain, which comprises a cysteine-rich subdomain that forms disulfide bridges and a tyrosinase-like subdomain responsible for binding bimetal ions. Indeed, the tyrosinase-like subdomain of TYR contains two Cu atoms, whereas TRP-1 and DCT are zinc (Zn)-containing enzymes. In these enzymes, the metal ions are coordinated by six histidine residues within the active site cavity [27,28]. However, the conformational flexibility of substrates and intermediate products during melanin biosynthesis varies in relation to the TYR family enzymes, depending on their orientation and distance from the metal ions. To illustrate, molecular docking simulations revealed that the substrates such as L-tyrosine, L-DOPA, DHI, and DHICA bind to the recombinant TYR enzyme in suitable orientations. In contrast, ligand DQ was found to bind the enzyme in inappropriate positions [29].

TYR plays a key role as the rate-limiting enzyme in melanogenesis. It catalyzes both monophenolase and diphenolase reactions. In this study, we determined the inhibitory effects of the samples on monophenolase activity by measuring the final product, L-dopachrome, formed from a mixture of the tested samples, L-tyrosine, and mushroom tyrosinase. For diphenolase activity, the reaction involved mixing the tested samples with L-DOPA and mushroom tyrosinase. The results indicated that 95EtOH demonstrated the greatest inhibitory effects on both monophenolase and diphenolase activities of mushroom tyrosinase (Table 2). This effect may be attributed to its phenolic content, especially rosmarinic acid, the main compound in the 95EtOH banana peel extract.

Furthermore, molecular docking analysis with mushroom tyrosinase (PDB ID: 2Y9X) deriving from mushroom *A. bisporus* showed that *(R)*-rosmarinic acid exhibited a strong binding affinity to this enzyme, with a binding energy of −5.05 kcal/mol. Regarding the binding energies of the substrates, L-tyrosine and L-DOPA towards tyrosinase enzyme were found to be −5.18 and −5.37 kcal/mol, respectively (Table 3). Additionally, we found that rosmarinic acid interacted with *A. bisporus* tyrosinase at the same binding sites as L-tyrosine and L-DOPA, including HIS263, VAL283, and MET280. As for the binding interactions between the tyrosinase inhibitors, β-arbutin and kojic acid, with mushroom tyrosinase, VAL283 and MET280 were also found to form similar bonds with the target enzyme (Figure 1). These results were consistent with a previous study, which showed that kojic acid established essential bonds with the binding pocket of 2Y9X at GLY281, HIS61, HIS85, HIS263, VAL283, MET280, ASN60, and ALA286 [30]. Despite the results from another binding model towards 2Y9X, the predominant interactions between arbutin and the active site of tyrosinase included HIS85, HIS263, and ASN280. Both rosmarinic acid and arbutin were located at the tropolone binding site of 2Y9X at the same position HIS263. Moreover, rosmarinic acid exhibited more hydrogen bonding with ASN260 and VAL283 [31]. Along with another molecular simulation study, these findings confirmed that the interactions of ligands with MET280, VAL283, and HIS85 through hydrogen bonding, or with HIS263 or VAL283 via π-bonding within the tyrosinase cavity, can influence changes in enzyme conformation [32]. In our study, the aromatic ring of rosmarinic acid was found to bind to *A. bisporus* tyrosinase at HIS263 and VAL283, forming an additional π-π stacked bond with SER282 (Figure 2).

In this study, we further conducted anti-melanogenesis assays using both human and mouse melanoma cells to confirm the effects of banana peel extracts and their bioactive compounds. Our results illustrated that all banana peel extracts could inhibit melanin content as well as cellular tyrosinase activity in IBMX-induced B16 melanoma cells. In contrast, in G361 human melanoma cells, 95EtOH significantly reduced intracellular tyrosinase activity, consistent with the effects observed in the tyrosinase inhibitor treatment groups (Figure 4). Rosmarinic acid is the predominant compound found in all extracts. Several studies have reported that rosmarinic acid possesses strong antioxidant potential, scavenging oxidative stress and preventing pigmentation by disrupting the formation of intermediate products during melanin biosynthesis. Rosmarinic acid and its derivatives can decrease the level of melanin produced in B16 mouse melanoma cells [31,32]. Furthermore, in human melanoma A375 cells, rosmarinic acid treatment has been shown to reduce cell proliferation, migration, and melanin production by blocking glycogen synthase kinase-3β (GSK-3β) [33]. Similarly, in a study conducted on female volunteers affected with dark facial spots, the topical application of a formulation containing dextran conjugated with rosmarinic acid for 2 months increased skin radiance, as indicated by elevated ITA° values [34]. Alongside this, ferulic acid, also present in 50EtOH, exhibits antioxidant properties that inhibit melanocyte proliferation [14].

The activation of the *MITF* transcription factor gene controls melanocyte pigmentation by inducing pigmentary gene expression, involving genes such as *TYR*, *TRP-1*, and *DCT*. It is well established that exposure to cAMP-elevating agents, such as IBMX or forskolin, can upregulate *MITF* expression, leading to increased melanin synthesis [2,35]. In the results obtained from B16 melanoma cells, we found that all extracts reduced the relative expression of target genes associated with melanogenesis, alongside the phenolic compounds ferulic acid and rosmarinic acid. In the case of human melanoma cells, the ethanolic extracts of banana peels (50EtOH and 95EtOH) displayed more effective inhibitor effects on melanogenesis-related genes than treatment with phenolic compounds alone (Figure 5). These effects may be attributed to other groups of phytochemical constituents. Our results were consistent with those from other banana species, whose peels were rich in phenolic compounds. The formulation containing unripe peels of *M. sapientum* ABB group ‘Kluai Namwa’ extract exhibited inhibitory effects against the diphenolase activity of mushroom tyrosinase [21]. In the case of the peel extract from *M. sapientum* AA group ‘Leb Mu Nang’, the finding suggested that this extract could inhibit melanin content in B16 melanoma cells by downregulating the protein expression of MITF, TYR, and melanosome transfer markers, including Ras-related protein (Rab) 27a and premelanosome protein 17 (Pmel17) [22]. Additionally, sucrier banana peel also demonstrated significant inhibition of melanogenesis through the p38 signaling pathway in B16 cells [23]. Interestingly, mice fed the fruit of *M. sapientum* AA group ‘Khai’ exhibited reduced oxidative stress in their skin after exposure to UVB radiation [36].

Considering the above, peel extracts of Hom Thong banana species could be effective for application on hyperpigmented skin lesions due to their inhibitory effects on tyrosinase. These effects are primarily attributed to their phenolic compounds, particularly rosmarinic acid, which is predominantly found in 95EtOH. Our findings confirmed that these extracts could reduce melanin content and L-dopachrome formation by inhibiting the regulator gene *MITF*, as well as genes related to melanin biosynthesis, including *TYR*, *TRP-1*, and *DCT*. However, other groups of bioactive compounds that remain unidentified in these extracts may also contribute to the observed effects on melanogenesis inhibition. Hence, our future research will focus on enhancing the release of phytochemical compounds using ultrasonic or microwave assistance to rapture plant cell walls with minimal impact on the stability of bioactive compounds. Additionally, we will identify these additional bioactive compounds using methods such as Quadrupole Time-of-Flight Mass Spectrometry (QTOF) analysis and study their effects on melanogenesis. Moreover, further studies will include additional models, such as co-culture systems, to elucidate the interactions between melanoma cells and surrounding skin cells, such as keratinocytes and fibroblast cells. This study does not address the long-term effects or potential side effects associated with the use of Hom Thong banana peel extracts. Future studies should focus on determining the optimal concentration for incorporation into topical formulations through irritation assays using an ex vivo model to evaluate irritation levels. Furthermore, clinical studies may also be conducted to confirm the efficacy and safety of these extracts for long-term applications.

## 4. Materials and Methods

### 4.1. Plant Materials and Extracts Preparation

Ripe bananas (*Musa* sp., AAA group; Thai accession ‘Hom Thong’) were purchased from the local market in Lamphun Province, Northern Thailand, in June 2023. Voucher samples were identified and deposited at the herbarium of the Pharmaceutical and Natural Products Research and Development Unit (PNPRDU), number PNPRDU66001. Dry peels (2000 g each) were powdered and then separately extracted with solvents in a ratio of 1:2 *w*/*v* (distilled water, 50% ethanol, or 95% ethanol). The extract solution was filtered through Whatman filter paper no. 1. Afterward, the solvent was removed by a freeze-dryer (Beta 2–8 LD-plus, Martin Christ Gefriertrocknungsanlagen GmbH, Osterode am Harz, Germany) for aqueous extraction or a rotary evaporator (Hei-VAP value, Heidolph, Schwabach, Germany) for ethanolic extraction [37]. Aqueous, 50% ethanolic, and 95% ethanolic extracts of banana peels were labeled as Water, 50EtOH, and 95EtOH, respectively. All samples were stored at 4 °C until they were analyzed further.

### 4.2. Determination of Phenolic Profiles Using Liquid Chromatography Coupled with Electrospray Ion Quadrupole Mass Spectrometry (LC-ESI/MS)

The polyphenol profiles analysis was conducted following the methodology of a previous study [35], using an Agilent 1260 Infinity II series, coupled with an ESI quadrupole MS 6130 detector (Agilent Technologies, Santa Clara, CA, USA). Chromatographic separation was carried out using a Restek Ultra C18 reverse-phase column (250 × 4.6 mm, 5 µm, Restek, Bellefonte, PA, USA). The mobile phases consisted of 0.2% acetic acid in 95% water and 5% methanol (Solvent A) and 0.2% acetic acid in 50% water and 50% acetonitrile (Solvent B). A linear gradient elution of the mobile phases was applied as follows: 10–20% Solvent B from 0 to 45 min, 20–55% B from 45 to 85 min, 55–100% B from 85 to 97 min, and 100% B from 97 to 110 min. The column temperature was maintained at 40 °C in gradient mode. Samples were injected at a volume of 20 μL with a flow rate of 0.5 mL/min. Spectra were acquired in negative selected ion monitoring (SIM) mode, with capillary and nozzle voltages set to −3.5 V. Dry nitrogen gas was used for nebulization at a flow rate of 12 L/min, with temperature of 250 °C in the dissolving line and 400 °C in the block source. A fragmentor voltage of 70 V was applied, and full mass scans were performed in the range of m/z 100 to 1200, with a scan speed of 250 ms per spectrum. The resulting spectra were processed using OpenLab CDS software Rev.C.01.07 SR3 (465) (Agilent Tech., Santa Clara, CA, USA).

### 4.3. Mushroom Tyrosinase Activity Assay

Mushroom tyrosinase enzyme was used to screen the tyrosinase inhibitory activity of banana peel extracts according to a previous protocol [38]. Mushroom tyrosinase from *A. bisporus* (T3824, Sigma Chemical, St. Louis, MO, USA) was prepared at the concentration of 100 units/mL in 0.1 M phosphate buffer. Extract solutions of *Musa* sp. (40 µL) were individually mixed with 80 µL of phosphate buffer and 40 µL of mushroom tyrosinase. Subsequently, the substrate (40 µL) was added to the reaction mixture, and absorbance measurement began after 30 min of incubation in the dark at room temperature. The monophenolase activity of tyrosinase was measured using L-tyrosine (Bio Basic, Markham, ON, Canada) as the substrate. The substrate L-DOPA (Sigma Chemical, St. Louis, MO, USA) was used to determine the diphenolase activity of tyrosinase. The absorbance of L-dopachrome formation was detected at 475 nm. Mushroom tyrosinase inhibition was calculated using Equation (1):

Mushroom tyrosinase inhibition (%) = (A–B) − (C–D)/(A–B) × 100(1)

where A, B, C, and D denote the vehicle control, vehicle control without mushroom tyrosinase, extract solution mixed with mushroom tyrosinase, and extract solution without mushroom tyrosinase, respectively.

The concentration and the percentage of mushroom tyrosinase inhibition of each sample were plotted to obtain the linear equation. The concentration of each sample that resulted in 50% mushroom tyrosinase inhibition was recognized as the IC_50_ value.

### 4.4. Computational Study

#### 4.4.1. Protein and Ligands Preparation

X-ray crystal structure of *A. bisporus* mushroom tyrosinase in complex with the specific tyrosinase inhibitor, tropolone, was retrieved from the RSCB Protein Data Bank (PDB ID: 2Y9X) [8]. The protein structure was prepared using Discovery Studio 2.5 software (Discovery, New York, NY, USA), selecting only chain A (which contains the H subunit with a binuclear Cu-binding site). Tropolone and all hetero atoms, except for two Cu ions, were removed. The tyrosinase structure was then cleaned, and hydrogen atoms were added. The three-dimensional structures of all ligands, including natural substrates, tyrosinase inhibitors, and phenolic compounds identified from Hom Thong banana peel extracts, were retrieved from the PubChem database. All ligands’ 3D structures, except for the native tropolone, were optimized using the Gaussian 16 program (Gaussian Inc., Wallingford, CT, USA) with the B3LYP model and a 6–31G (d, p) basis set [39].

#### 4.4.2. Molecular Docking Study for Mushroom Tyrosinase

Tropolone was re-docked into the tyrosinase active site on the H-subunit for the method validation. Subsequently, the substrates L-tyrosine and L-DOPA, positive controls β-arbutin and kojic acid, along with phenolic compounds including (+)-catechin, *trans*-*o*-coumaric acid, *trans*-ferulic acid, and *(R)*-rosmarinic acid, were docked into the tyrosinase active site. All docking simulations were performed using AutoDock 4.2.6 and AutoDockTools, the graphical user interface [40]. For all dockings, the grid box dimensions (x, y, z) were set as 40, 40, 40 Å, with a grid spacing of 0.375 Å. The grid center was set at −10.215, −28.653, −43.444 (XYZ coordinate) within the active site. The “AD4_parameters.dat” file in AutoDock was manually edited to include Cu ion parameters (arbitrary charge of +2, Rii = 3.50 Å, epsii = 0.005 kcal/mol and vol = 12 Å^3^) [41,42]. The Lamarckian genetic algorithm was used to search for 100 conformations, starting from an initial population of 300 random conformations. Each run was set to terminate upon meeting the two stop criteria: a maximum of 2,500,000 energy evaluations and a maximum of 27,000 generations. Default settings for the reference root-mean-square were maintained, with a crossover rate of 0.8 and a mutation rate of 0.02. Docked poses were clustered and sorted based on binding energy, with an RMSD tolerance of 2 Å. The docked conformation with the most negative binding free energy from the most crowded cluster was selected for interaction and binding mode analysis using BIOVIA Discovery Studio Visualizer and the PyMOL (Schrödinger, Inc., New York, NY, USA) [43].

### 4.5. Cell Culture and Cell Viability Assay

G361 human melanoma (IFO50009) and B16 mouse melanoma (JCRB0202) cells were obtained from the JCRB cell bank (Osaka, Japan). G361 and B16 melanoma cells were grown in Eagle’s minimal essential medium (Gibco, Grand Island, NY, USA) supplemented with 10% fetal bovine serum (HyClone™ Cytiva, Pasching, Austria) at 37 °C with 5% CO_2_. All cells from passages 3 to 10 were used in the tests. The Sulforhodamine B (SRB) assay was used to determine the cytotoxicity effects of banana peel extracts, as previously reported [44]. The cells were seeded and exposed to different concentrations of extracts (0.03125–1 mg/mL) for 48 h and 72 h. After incubation, the medium was discarded and replaced with 50% trichloroacetic acid (PanReac AppliChem, Barcelona, Spain) to fix the cells. The plate was washed with distilled water three times and left to dry overnight. The 0.04% SRB solution (Sigma Chemical, St. Louis, MO, USA) was added to each well for staining. The unbound dye was removed and washed with 1% glacial acetic acid. The stained dye was solubilized using a 10 mM tris base solution (Vivantis, Shah Alam, Selangor, Malaysia). The absorbance was determined at a wavelength of 515 nm. The relative percentage of cell viability was calculated compared to untreated control cells. Tested concentrations with cell viability above 80% will be chosen for the following experiments.

### 4.6. Determination of Intracellular Melanin Content

A melanin content assay was performed to investigate the amount of melanin produced in melanoma cells [45]. In brief, cells were seeded in 6-well plates and incubated overnight. Cells were pre-treated with banana peel extracts for 1 h and further induced with 100 μM 3-isobutyl-1-methylxanthine (IBMX, Sigma Aldrich, St. Louis, MO, USA) for an additional 48 h. The medium was removed, and wells were washed with phosphate-buffered saline. Cells were harvested and collected cell pellets to react with an alkaline solution (1N sodium hydroxide) to dissolve the intracellular melanin in the cells. After 1 h of incubation, the mixture solution was determined at a wavelength of soluble melanin at 405 nm. The relative percentage of melanin level was calculated compared to untreated control cells.

### 4.7. Determination of Intracellular Tyrosinase Activity

L-DOPA was used as a substrate for the intracellular tyrosinase activity assay. The tyrosinase activity was quantified indirectly by measuring the amount of L-dopachrome formation within the cells, using a method from a previous study [46]. In brief, after treatment, harvested cells were lysed in 1% Triton X-100 (VWR Life Science, Solon, OH, USA). The cell supernatant was then reacted with 5 mM L-DOPA. After incubation, the reaction was measured at a wavelength of 475 nm for dopachrome formation. The relative percentage of tyrosinase activity was calculated compared to untreated control cells.

### 4.8. Determination of Pigmentary Genes Expression

Total RNA was extracted using the E.Z.N.A.^®^ Total RNA Kit I (Omega Bio-Tek, Norcross, GA, USA) according to the manufacturer’s procedure. The method was carried out following a previously published protocol [46]. The RNA concentration was quantified using the NanoDrop^TM^ One^C^ Microvolume UV-Vis Spectrophotometer (Thermo Fisher Scientific, Waltham, MA, USA). Reverse transcription was performed using the MyTaq^TM^ One-Step RT-PCR Kit (Meridian Bioscience^TM^, BIO-65049, Memphis, TN, USA). Nucleic acid was amplified using the T100^TM^ Thermal Cycler (Bio-Rad, Hercules, CA, USA). The primer sequences, along with *GAPDH*, which is used as a control for normalization, are listed in Table 4. RT-PCR products were loaded onto 1% agarose gels stained with ViSafe Red (Vivantis, Shah Alam, Selangor, Malaysia). The bands were separated in the gels by electrophoresis and measured using the Gel Doc^TM^ EZ System and Image Lab^TM^ software 6.1 (Bio-Rad, Hercules, CA, USA).

### 4.9. Statistical Analysis

All data are presented as mean ± standard deviation. Statistical analysis was conducted using one-way ANOVA followed by the LSD post hoc test (SPSS 23.0 software, Chicago, IL, USA). A significance level of *p* < 0.05 was considered statistically significant.

## 5. Conclusions

Crude extracts of banana peels from *Musa* sp., AAA group ‘Hom Thong’, comprised phenolic profiles including catechin, *o*-coumaric acid, ferulic acid, and rosmarinic acid. Notably, rosmarinic acid is the main phenolic compound found in all extracts, Water, 50EtOH, and 95EtOH. To screen for melanogenesis inhibition, we determined the levels of L-dopachrome formation through reactions involving samples, substrates (L-tyrosine or L-DOPA), and mushroom tyrosinase enzyme. According to our results, 95EtOH demonstrated the strongest inhibitory effects against the monophenolase and diphenolase activities of mushroom tyrosinase. To elucidate the interaction between the bioactive compounds and the tyrosinase target enzyme, we conducted a molecular docking study using the crystal structure of *Agaricus bisporus* mushroom tyrosinase (PDB ID: 2Y9X). Our findings suggested that natural substrates, including L-tyrosine or L-DOPA, bind to tyrosinase via π-bonds at residues HIS263 and VAL283. Moreover, rosmarinic acid formed bonds similar to those of the substrates, with an additional interaction at residue SER282. To confirm these effects, we performed anti-melanogenesis assays in both human and mouse melanoma cells. We found that all extracts and their bioactive compounds significantly inhibited melanin content and tyrosinase activity in B16 mouse melanoma cells stimulated with IBMX. However, in the G361 human melanoma cells, 95EtOH showed the most effective inhibitory effects against melanin production and intracellular tyrosinase activity. These effects were consistent with the results of the determination of gene expression related to melanin biosynthesis, involving genes such as the regulator gene *MITF*, the rate-limiting enzyme tyrosinase encoded by *TYR*, and tyrosinase family members including *TRP-1* and *DCT*. Therefore, banana peel extracts from Hom Thong species could be applied as depigmenting agents.

## Figures and Tables

**Table 1:** Phenolic profiles of three types of Hom Thong banana peel extracts.

Bioactive Components (mg/g Extract)	Water	50EtOH	95EtOH
Catechin	1.89 ± 0.05	ND	ND
*o*-Coumaric acid	0.65 ± 0.01	ND	ND
Ferulic acid	ND	0.21 ± 0.00	ND
Rosmarinic acid	0.12 ± 0.00 ^a^	0.55 ± 0.02 ^b^	0.90 ± 0.00 ^c^

Data are expressed as the mean ± SD. Significant differences between samples are indicated by different letters (a, b, and c) with *p* < 0.05. Water: aqueous extract of Hom Thong banana peel, 50EtOH: 50% ethanolic extract of Hom Thong banana peel, 95EtOH: 95% ethanolic extract of Hom Thong banana peel, ND: Not detected.

**Table 2:** Inhibitory effects of standard tyrosinase inhibitors, Hom Thong banana peel extracts, and their bioactive compounds on mushroom tyrosinase activity.

Samples	IC_50_ Values (mg/mL)					
	L-Tyrosine as the Substrate			L-DOPA as the Substrate		
Water	5.56	±	0.46 ^f^	17.35	±	3.24 ^d^
50EtOH	2.33	±	0.23 ^c^	2.70	±	0.07 ^ab^
95EtOH	0.92	±	0.15 ^a^	2.01	±	0.17 ^a^
Arbutin	1.09	±	0.03 ^ab^	3.44	±	0.04 ^b^
Kojic acid	4.66	±	0.05 ^e^	3.48	±	0.18 ^b^
Catechin	4.97	±	0.07 ^e^	6.42	±	0.45 ^c^
*o*-Coumaric acid	1.39	±	0.03 ^b^	4.19	±	0.15 ^b^
Ferulic acid	3.54	±	0.10 ^d^	3.93	±	0.09 ^b^
Rosmarinic acid	3.32	±	0.12 ^d^	3.62	±	0.08 ^b^

Data are expressed as the mean ± SD. Significant differences between samples are indicated by different letters (a, b, c, d, e, and f) with *p* < 0.05. Water: aqueous extract of Hom Thong banana peel, 50EtOH: 50% ethanolic extract of Hom Thong banana peel, 95EtOH: 95% ethanolic extract of Hom Thong banana peel.

**Table 3:** Binding free energy of L-tyrosine, L-DOPA, and bioactive compounds found in Hom Thong banana peel extracts with mushroom tyrosinase.

Ligands	PubChem CID	Binding Free Energy, ∆G (kcal/mol)
L-DOPA	6047	−5.37
L-Tyrosine	6057	−5.18
(+)-Catechin	9064	−5.07
*(R)*-Rosmarinic acid	5281792	−5.05
β-Arbutin	440936	−4.06
Kojic acid	3840	−3.60
*trans*-*o*-Coumaric acid	637540	−3.34
*trans*-Ferulic acid	445858	−3.10

**Table 4:** List of primer sequences used for gene expression analysis.

Target	Species	Forward Primer (5′ to 3′)	Reverse Primer (5′ to 3′)	References
*MITF*	Human	ACCGTCTCTCACTGGATTGGT	ACCAATCCAGTGAGAGACGGT	[45]
	Mouse	ATCCCATCCACCGGTCTCTG	CAGAGACCGGTGGATGGGAT	[47]
*TYR*	Human	TTGGCATAGACTCTTCTTGTTGCGG	CCGCAACAAGAAGAGTCTATGCCAA	[45]
	Mouse	AAGAATGCTGCCCACCATGG	CCATGGTGGGCAGCATTCTT	[47]
*TRP-1*	Human	TGGCAAAGCGCACAACTCACCC	GGGTGAGTTGTGCGCTTTGCCA	[45]
	Mouse	CAGTGCAGCGTCTTCCTGAG	CTCAGGAAGACGCTGCACTG	[47]
*DCT*	Human	TGTGGAGACTGCAAGTTTGGC	GCCAAACTTGCAGTCTCCACA	[45]
	Mouse	GATGGCGTGCTGAACAAGGA	TCCTTGTTCAGCACGCCATC	[47]
*GAPDH*	Human	GGAAGGTGAAGGTCGGAGTC	CTCAGCCTTGACGGTGCCATG	[45]
	Mouse	CCTCGTCCCGTAGACAAAATG	CATTTTGTCTACGGGACGAGG	[48]

## Data Availability

The data contributions presented in this study are included in the article and the Supplementary Materials.

## Supplementary Materials

The following supporting information can be downloaded at: https://www.mdpi.com/article/10.3390/ijms252313202/s1.
