# Supplementary material for: Valorization of Hom Thong Banana Peel (Musa sp., AAA Group) as an Anti-Melanogenic Agent Through Inhibition of Pigmentary Genes and Molecular Docking Study
Source: Int J Mol Sci. 2024 Dec 8;25(23):13202. doi: 10.3390/ijms252313202 (PMC11642236; doi:10.3390/ijms252313202)
Supplement: Supplementary file 1 [file ijms-25-13202-s001.zip › ijms-3328245-supplementary.pdf]

Article

Valorization of Hom Thong Banana Peel (*Musa* sp., AAA Group) as an Anti-Melanogenic Agent Through Inhibition of Pigmentary Genes and Molecular Docking Study

Supplementary Information

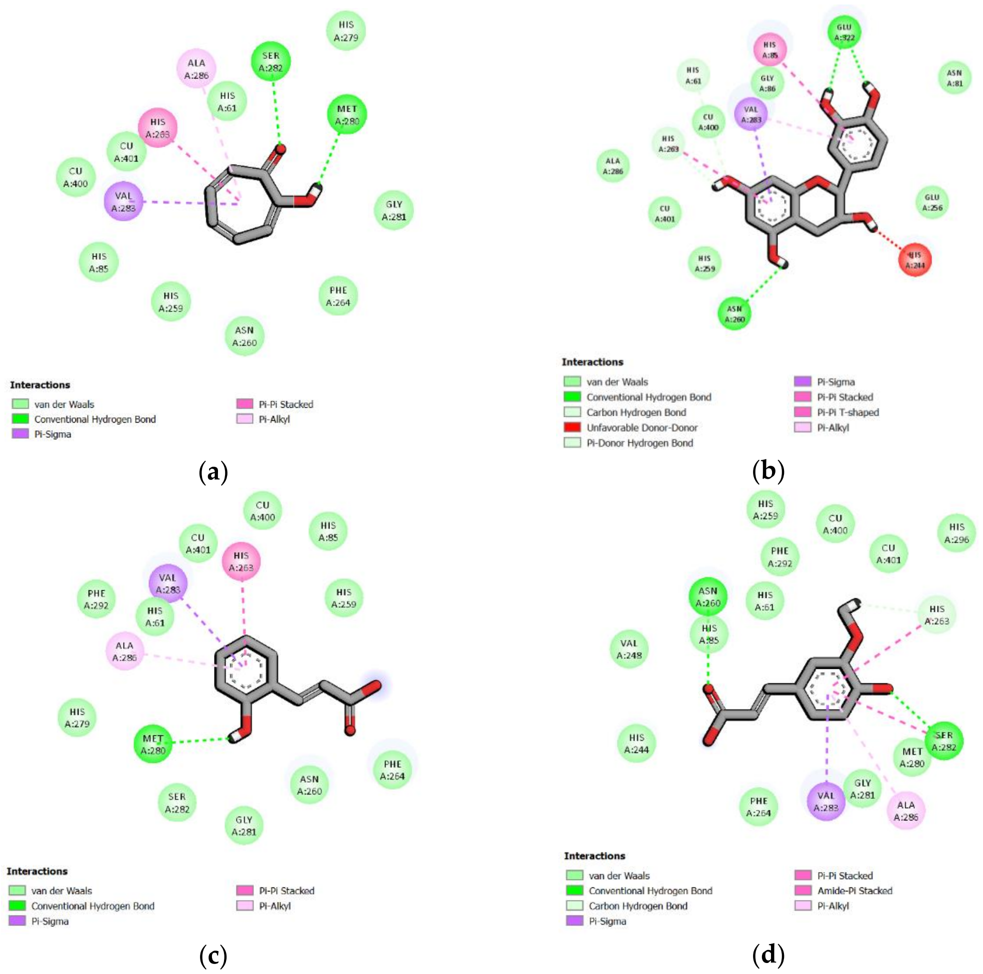

Figure S1. The molecular interaction profile of mushroom tyrosinase (PDB ID: 2Y9X) and the ligands after molecular docking studies binding poses of (**a**) tropolone; (**b**) (+)-catechin; (**c**) *trans*-*o*-coumaric acid; and (**d**) *trans*-ferulic acid visualized by the BIOVIA Discovery Studio Visualizer.

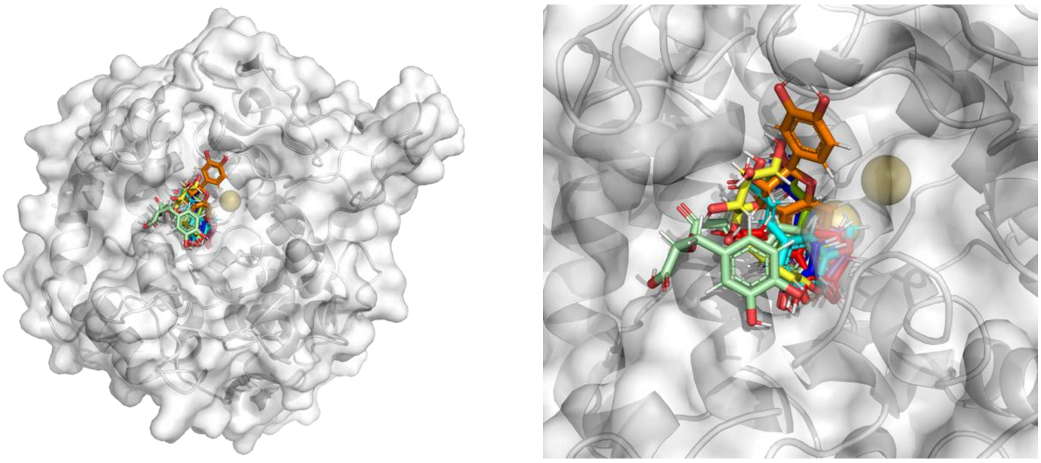

(**a**) (**b**)

Figure S2. Superimpose of the highest negative binding free energy conformation of each ligand in the active site of mushroom tyrosinase (PDB ID: 2Y9X). Ligands are shown in various colors in the stick mode. Backbone receptor is shown in white color with the cartoon and surface form while the Cu atoms are shown in yellow-orange sphere.
